# The CSB chromatin remodeler and CTCF architectural protein cooperate in response to oxidative stress

**DOI:** 10.1093/nar/gkv1219

**Published:** 2015-11-17

**Authors:** Robert J. Lake, Erica L. Boetefuer, Kyoung-Jae Won, Hua-Ying Fan

**Affiliations:** 1 Epigenetics Program, Perelman School of Medicine, University of Pennsylvania, Philadelphia, PA 19104,USA; 2 Department of Biochemistry and Biophysics, Perelman School of Medicine, University of Pennsylvania, Philadelphia, PA 19104, USA; 3 Biology Graduate Program, Graduate School of Arts and Sciences, University of Pennsylvania, Philadelphia, PA 19104, USA; 4 Institute for Diabetes Obesity and Metabolism, Perelman School of Medicine, University of Pennsylvania, Philadelphia, PA 19104, USA; 5 Department of Genetics, Perelman School of Medicine, University of Pennsylvania, Philadelphia, PA 19104, USA

## Abstract

Cockayne syndrome is a premature aging disease associated with numerous developmental and neurological abnormalities, and elevated levels of reactive oxygen species have been found in cells derived from Cockayne syndrome patients. The majority of Cockayne syndrome cases contain mutations in the ATP-dependent chromatin remodeler CSB; however, how CSB protects cells from oxidative stress remains largely unclear. Here, we demonstrate that oxidative stress alters the genomic occupancy of the CSB protein and increases CSB occupancy at promoters. Additionally, we found that the long-range chromatin-structure regulator CTCF plays a pivotal role in regulating sites of genomic CSB occupancy upon oxidative stress. We show that CSB directly interacts with CTCF *in vitro* and that oxidative stress enhances the CSB-CTCF interaction in cells. Reciprocally, we demonstrate that CSB facilitates CTCF-DNA interactions *in vitro* and regulates CTCF-chromatin interactions in oxidatively stressed cells. Together, our results indicate that CSB and CTCF can regulate each other's chromatin association, thereby modulating chromatin structure and coordinating gene expression in response to oxidative stress.

## INTRODUCTION

Reactive oxygen species (ROS) are constantly generated during aerobic metabolism. When ROS overloads the cellular antioxidant defense systems, the resulting alteration in redox homeostasis leads to oxidative stress (1). Oxidative stress has been implicated in the aging process and diseases, such as cancer and neurological disorders. Cockayne syndrome is a premature aging disease associated with neurological and developmental abnormalities as well as sun sensitivity (2). Although the underlying mechanisms that lead to the diverse features of Cockayne syndrome remain largely unknown, a reduced ability of cells to relieve oxidative stress has been proposed to be a leading cause (3–5).

Mutations in the Cockayne syndrome group B protein (CSB) account for the majority of Cockayne syndrome cases (6). CSB belongs to the SWI2/SNF2 ATP-dependent chromatin remodeler family, which is conserved from yeast to human (7). These proteins alter chromatin structure in an ATP-dependent manner and regulate fundamental nuclear processes, such as transcription and DNA repair. CSB displays ATP-dependent chromatin remodeling activities *in vitro* and in cells (8–10).

CSB functions in transcription regulation, in addition to its better-characterized function in transcription-coupled nucleotide excision repair (11,12). Transcription profiling assays have indicated that CSB plays a general role in transcription regulation (11,13), and a direct role of CSB in transcription regulation was demonstrated by identifying genomic occupancy sites of the CSB protein. CSB is enriched at regions with epigenomic features of promoters and enhancers (9). Importantly, CSB alters nucleosome structure near its occupancy sites to directly regulate gene expression (9).

Upon oxidative stress, CSB-deficient cells display increased cell death as compared to CSB-expressing cells (3,14,15). Increased ROS levels, altered gene expression and damaged DNA are observed in primary cells, iPS cells and immortalized cells derived from Cockayne syndrome patients (4,11,16–18). To understand further how CSB relieves oxidative stress, we identified sites of genomic CSB occupancy upon oxidative stress using chromatin immunoprecipitation followed by deep sequencing (ChIP-seq). We found that CSB co-localizes with CTCF, a CCCTC-binding transcription factor and a major regulator of long-range chromatin interactions (19), at a subset of genomic regions upon oxidative stress. We also found that CSB and CTCF directly interact and can regulate each other's chromatin association in response to oxidative stress.

## MATERIALS AND METHODS

### Cell culture and menadione treatment

CS1AN-sv cells and CS1AN-sv cells stably expressing CSB were maintained in DMEM-F12 supplemented with 10% FBS (6,8,9). For the ChIP-seq, ChIP-qPCR and co-IP assays, oxidative stress was induced by treating cells with 100 μM menadione in culture medium for 1 hour. For the cell survival and protein-fractionation assays, menadione concentrations are as noted in the text and figures.

### Protein fractionation

Equal numbers of cells were seeded onto five 60 mm dishes and allowed to grow overnight until ∼80% confluent. Cells were treated with varying concentrations of menadione in growth medium for 1 h or left untreated. Cells were then rinsed with PBS and collected in 200 μl buffer B (150 mM NaCl, 0.5 mM MgCl_2_, 20 mM HEPES (pH 8.0), 10% glycerol, 0.5% Triton X-100, 1 mM DTT) on ice, as described previously (20). Cell lysates were centrifuged at 20 000 × g for 20 min at 4°C, and 150 μl supernatant was added to 50 μl 4× SDS sample buffer; this was the soluble fraction (S). 200 μl 1× SDS sample buffer was added to the pellet, which was then sonicated for 10 s at 25% amplitude using a Branson 101-135-126 Sonifier; this was the chromatin-enriched fraction (C). The resulting chromatin-enriched fractions were 1.3-fold more concentrated than the soluble fractions. 14 μl of each protein fraction was loaded on the gels. Antibodies used for western blot analysis were as described below. Western blots were developed using SuperSignal West Pico chemiluminescent substrate and imaged with a Fujifilm ImageQuant LAS-4000 imager.

To determine the percentage of CSB co-fractionating with chromatin, western blots were quantified using ImageJ. CSB signals were normalized to respective BRG1 signals. CSB co-fractionating with chromatin was calculated as ‘normalized CSB signals in ‘C’/(normalized CSB in ‘C’ + normalized CSB signal in ‘S’ x 1.3)’.

### shRNA knockdown

Mission lenti-viral shRNA expression constructs targeting CTCF (TRCN0000230191) and a non-targeting shRNA (SHC002) were purchased from Sigma-Aldrich. Virus was produced as previously described (9). The target cell confluence at the time of infection was ∼20%. Infected cells were harvested 5 days post-infection for chromatin immunoprecipitation (ChIP) and western blot analyses.

### ChIP-qPCR and ChIP-western analyses

ChIP was carried out following standard protocols. Briefly, 4-million cells were fixed with 1% formaldehyde for 10 min and sonicated on ice at 40% amplitude (30 s on, 90 s off, for a total of 24 min) using a Branson 101-135-126 Sonifier. ChIP was performed using 5 μl of a polyclonal anti-CTCF antibody (Millipore 07–729), 10 μl monoclonal anti-CSB antibody (1B1) (9) and 5 μl recombinant protein-G agarose beads (Invitrogen). ChIPed DNA was analyzed by real-time PCR using a 7900HT Fast Real-Time PCR System from Applied Biosystems and SYBR green. Primers were as described in Supplementary Table S7. For all ChIP-qPCR experiments described in this manuscript, menadione treated and untreated cells were examined side-by-side. For the ChIP-seq experiments, the CSB+M sample was processed alongside one untreated sample, which was previously reported (9).

For western blot analyses, ChIP samples were reverse cross-linked in SDS sample buffer at 95°C for 30 min (8).

### Antibodies

Antibodies used for western blot analysis were rabbit anti-CSB (1:2000) (21), rabbit anti-CTCF (1:2000) (Millipore, 07-729), mouse anti-GAPDH (1:10 000) (Millipore, MAB374), rabbit anti-BRG1 (1:1000) (22), rabbit anti-acetylated histone H3 (1:1000) (Millipore, 06-599), HRP-conjugated goat anti-rabbit IgG (1:10 000) (Pierce, 31460) and HRP-conjugated goat anti-mouse (IgG+IgM) (1:10 000) (Jackson Laboratory, 115-035-044).

### ChIP-Seq and data analysis

ChIP libraries for deep sequencing were constructed and sequenced as described previously (9). The resulting sequencing reads were mapped and peaks were identified as described in (9). Raw and processed files (GSE50925) have been deposited at the Gene Expression Omnibus (GEO) repository. CSB ChIP-seq data from untreated cells were previously published (GEO:GSE50171) (9).

ChIP sequencing reads within a 200 bp region around a peak center from the two cell populations were compared. If the difference between signal intensities was 4-fold or greater and the *P*-value for that difference was ≤0.0001, the peak was classified as ‘significantly induced by menadione’ (blue) ‘or’ significantly repressed by menadione (green)’ (Figure 2A). The remaining occupancy sites were classified as common (red) (Figure 2A).

The genomic distribution of CSB occupancy was classified using the gene annotation tool from UCSC RefGene as follows: (i) promoter (from −1 kb to the transcription start site), (ii) TTS (from the transcription termination site to +1 kb), (iii) 5′ UTR, (iv) 3′ UTR, (v) exon, (vi) intron and (vii) intergenic (the rest). The Genomic Regions Enrichment of Annotations Tool (GREAT, version 2.0.2) was used for pathway analysis of CSB occupancy sites, using the ‘MSigDB pathways’ category’ (23). The assignment of peaks to genes was made using the default setting (proximal 5 kb upstream and 1 kb downstream of a transcription start site, plus a distal extension to the regulatory elements of neighboring genes, up to 1000 kb) (23).

### Menadione sensitivity assays

100 000 cells were seeded onto 35 mm dishes. Twenty-four hours later, cells were either left untreated or treated with varying amounts of menadione in DMEM/F12 medium supplemented with 10% FBS for 1 h. After treatment, the menadione-containing medium was removed and fresh medium without menadione was added. The cells were subsequently cultured for an additional 24 h before cell viability was assayed. For cells infected with CTCF shRNA-expressing lentivirus, cells were treated with menadione 96 h post-infection, as described above, and assayed for survival 120 h post-infection. The number of viable cells was determined by trypan blue exclusion, using a hemocytometer. Percent survival was calculated as the ratio of treated cells to untreated cells.

### Constructs, protein expression and protein purification

CSB expression constructs were as previously described (20). For protein expression in SF9 cells, Flag-tagged proteins were purified using M2-affinity chromatography (22). MBP and MBP-CTCF (zinc fingers 1–11) were expressed and purified as described previously (24).

### *In vitro* protein–protein interaction assays

Purified, N-terminally Flag-tagged CSB, CSB-N, or CSB-C were incubated with MBP-CTCF immobilized on amylose beads at 4°C for 1 hour in PBS containing 0.5% Triton X-100 and 10 μM ZnSO_4_. The resulting amylose beads were washed with PBS + 0.5% Triton X-100 + 10 μM ZnSO_4_, and proteins were eluted in SDS sample buffer by heating beads at 95°C for 5 min.

### Gel shift assays

A 200 bp DNA fragment containing a CTCF-binding site was generated by PCR in the presence of ^32^P-dATP (Supplementary Figure S4B). Proteins were mixed with 1 nM ^32^P-labeled DNA at the indicated concentrations. Binding reactions were carried out in 30 mM HEPES (pH7.9), 60 mM NaCl, 6% glycerol, 6 mM MgCl_2_, 100 μM ZnSO_4_ and 0.02% NP40 at 30°C for 10 min. Reactions were loaded directly onto a 5% polyacrylamide gel prepared with 0.5× TBE. Gels were imaged using a Typhoon Trio (GE).

## RESULTS

### Oxidative stress induces changes in the genomic localization of CSB

**Figure 1.**
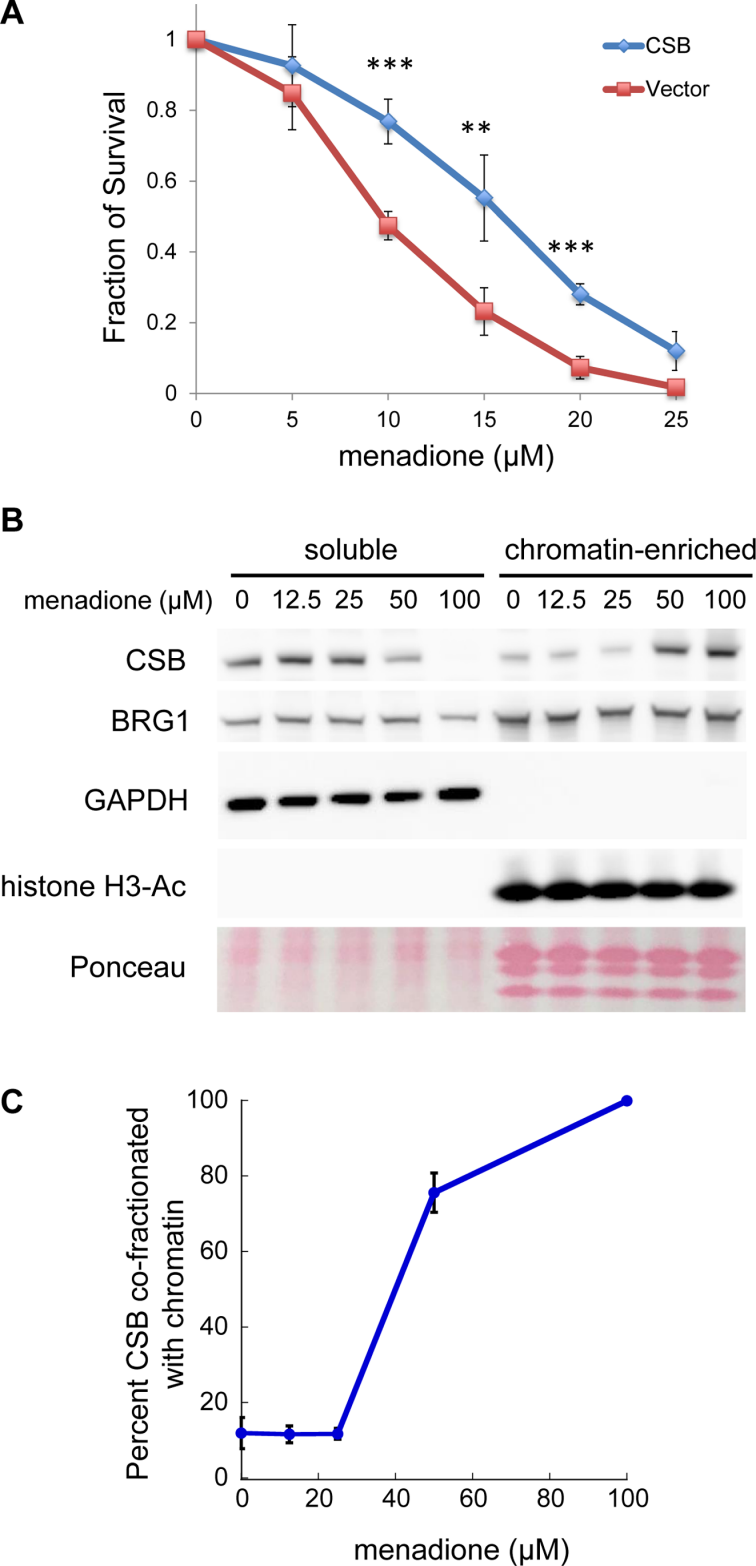
Menadione sensitivity assays. (**A**) CS1AN-sv cells were reconstituted with CSB^WT^ or an empty vector. Stable cell lines expressing transgenes were assayed for viability 24 h after a 1-h menadione treatment with the indicated menadione concentrations. Shown are means ± standard errors of the mean (SEM) (*n* = 5). A paired *t*-test was used to determine if the difference in menadione sensitivity of CS1AN cells before and after CSB add-back was significant. Triple asterisks indicate *P* values < 0.001, and double asterisks indicate *P* values < 0.01. (**B**) Analysis of CSB partitioning in cells after a 1-h menadione treatment, with menadione concentrations as indicated. Western blots were probed with antibodies as noted. BRG1 was used as a loading control. GAPDH and acetylated histone H3 were used as markers for soluble and chromatin-enriched fractions, respectively. Total core histones were visualized by Ponceau S staining. (**C**) Quantification of CSB levels in the soluble versus chromatin-enriched fraction. Shown are means ± SEM (*n* = 4).

To induce oxidative stress in cultured cells, we used menadione, which generates free radicals through redox cycling (25). To validate this system, we first determined if CS1AN-sv cells, which do not have functional CSB, were more sensitive to oxidative stress than CS1AN-sv cells reconstituted with CSB^WT^ (Figure 1A). As predicted, CS1AN-sv cells were more sensitive to menadione treatment than CSB-reconstituted CS1AN-sv cells.

We next determined if menadione treatment altered the CSB-chromatin interaction, using a fractionation protocol we have previously described (Figure 1B) (20). A 1-h menadione treatment at 50 μM and 100 μM induced the co-fractionation of CSB and chromatin, while this treatment did not have an apparent impact on another ATP-dependent chromatin remodeler, BRG1 (Figure 1B and C). As shown in Figure 1C, cells treated with 100 μM menadione for 1 h displayed a maximal increase in the amount of CSB co-fractionating with chromatin. Accordingly, we used 100 μM menadione to determine the genomic localization of CSB upon oxidative stress. Of note, menadione continuously generates reactive oxygen species (ROS) in cells through redox cycling (25). Consequently, in the cell-survival assays shown in Figure 1A, even though fresh medium was added to cells after one hour of menadione treatment, ROS can still be generated during the 24-h incubation in growth medium. Therefore, it is difficult to draw direct comparisons between menadione concentrations used in the survival assays (Figure 1A) to those used to induce CSB-chromatin co-fractionation (Figure 1B and C).

**Figure 2.**
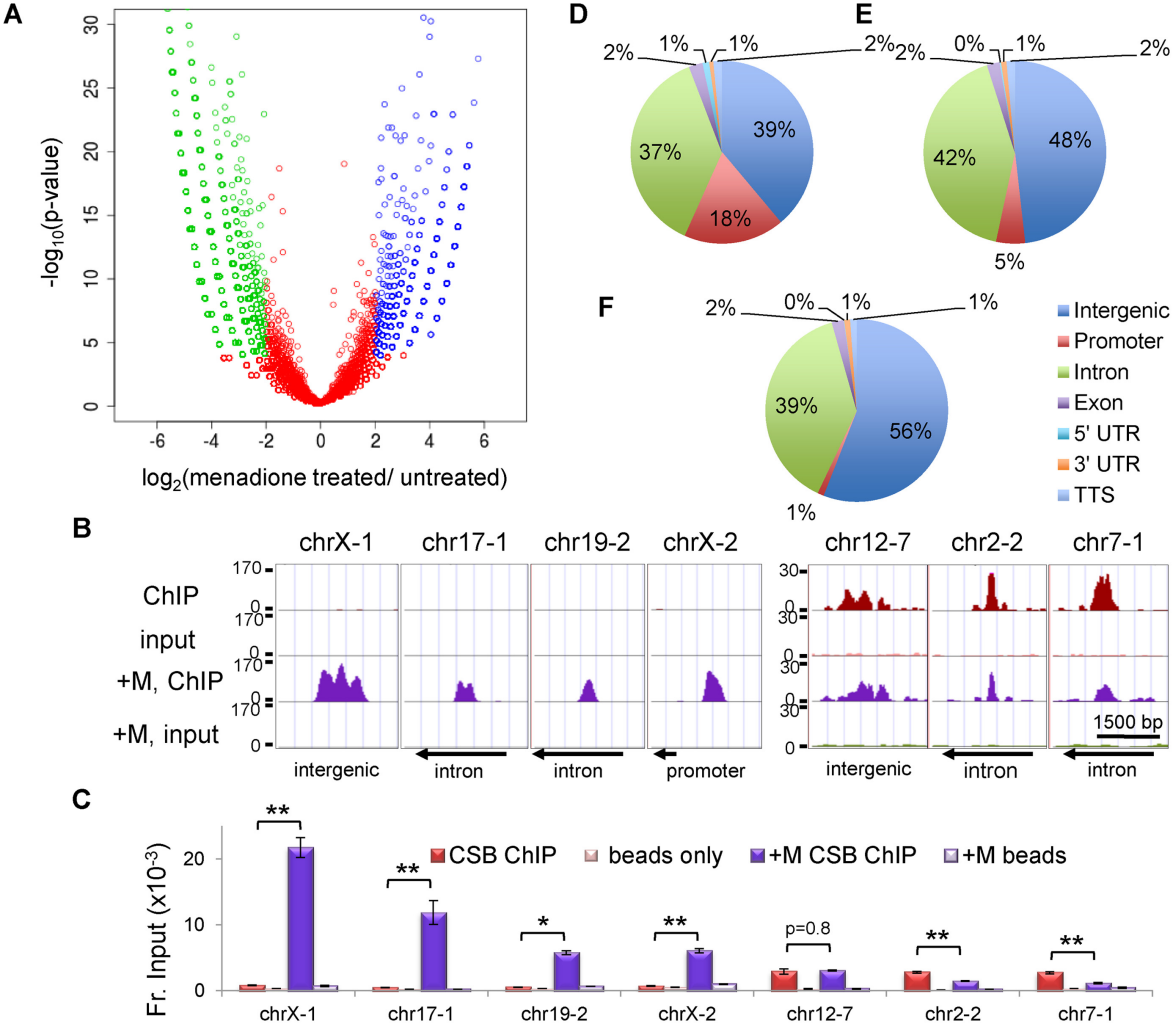
Comparison of CSB occupancy in cells with or without menadione treatment. (**A**) A volcano plot showing the correlation between CSB ChIP-seq results from cells with or without a 1-h menadione treatment (100 μM). (**B**) Screen shots of CSB ChIP-seq results from seven genomic regions, displayed using the UCSC genome browser. The y-axis is number of normalized sequencing reads. The x-axis represents the genomic coordinates; chrX-1, chrX:73766518–73766600; chr17-1, chr17:49770395–49770537; chr19-2, chr19:45793789–45793877; chrX-2, chrX:48568220–48568299, chr12-7, chr12:13679173–13679256; chr2-2, chr2:180325437–180325517; chr7-1, chr7:2001695–2001793. The directions of nearby transcription (arrows) are noted at the bottom. (**C**) Validation of CSB ChIP-seq results by ChIP-qPCR. Bar graphs showing CSB ChIP-qPCR results with matched beads-only controls. Shown are means ± SEM (*n* = 3). A paired t-test was used to determine if the difference in CSB enrichment before and after menadione treatment was significant. Single asterisks indicate *P* values < 0.05, and double asterisks indicate *P* values < 0.01. (**D**–**F**) Genomic distribution of CSB occupancy sites. The genome was divided into seven categories, as defined by the UCSC RefSeq gene annotation. (D) Menadione-induced CSB occupancy. (E) Common CSB occupancy. (F) Menadione-repressed CSB occupancy.

To determine the genomic localization of CSB upon oxidative stress, we performed CSB-ChIP-seq from cells treated with 100 μM menadione for 1 h. The resulting sequencing reads were mapped to the human genome, and peaks were identified using HOMER with a default option on ChIPed samples against matching input samples (26). In total, we recovered 19 063 CSB peaks in cells treated with menadione (Figure 2A, Supplementary Table S1).

We subsequently compared CSB occupancy in cells with or without menadione treatment. To do this, we compared signal intensities over a 200-bp region in cells treated with menadione to that in cells without treatment (9). If the difference between signal intensities was 4-fold or greater and the *P*-value for that difference was <0.0001, the signal was classified as menadione induced or repressed (blue or green, respectively, Figure 2A); the remaining signals were classified as common (red, Figure 2A). Among them, we identified 7070 CSB-occupancy sites induced by menadione treatment and 9163 CSB-occupancy sites repressed by menadione treatment, corresponding to ∼40% of total CSB-binding sites in each of the growth conditions (Figure 2A and Supplementary Tables S2–S4).

ChIP-qPCR was used to validate the ChIP-seq results at seven regions (Figure 2B and C). ChrX-1, chr17-1, chr19-2 and chrX-2 represent regions of menadione-induced CSB occupancy, and Chr12-7, chr2-2 and chr7-1 represent regions that are occupied by CSB but unaffected by menadione treatment (common). ChIP-qPCR confirmed that the occupancy of CSB at chrX-1, chr17-1, chr19-2 and chrX-2 was induced by menadione treatment: the increase in CSB enrichment at these sites in response to menadione was >4-fold, with *P*-value <0.01 (Figure 2C). CSB occupancy at chr12-7 was unaffected by menadione. Chr2-2 and chr7-1 were also occupied in both growth conditions, albeit with a slight decrease after menadione treatment. This slight reduction was significant, as the *P*-value was <0.01; however, these occupancy sites were considered common, as the signal intensities from the ChIP-seq results were within a 4-fold difference (Figure 2A and C).

### Promoter occupancy by CSB is increased upon oxidative stress

We then classified the CSB occupancy sites into seven functional categories, using the UCSC RefSeq gene annotations (Figure 2D–F). Previously, we found a modest but significant enrichment of CSB at promoter regions in unchallenged cells (9). Interestingly, upon menadione treatment, we observed further enrichment of CSB at promoters: from 2% of total CSB binding sites locating at promoter regions in untreated cells to 11% in menadione treated cells (*P*-value <1e−310 using Bernoulli's test) (Supplementary Figure S1A). Among CSB occupancy sites induced by a 1-h, 100 μM menadione treatment, 18% of them were located at promoters while the genomic distribution of promoters is only 1%. The fraction of promoter-occupied sites dropped to 5% among the ‘common’ peaks and 1% among the ‘repressed’ peaks (Figure 2D and E and Supplementary Tables S2–S4). These observations support a role of CSB in transcription regulation upon oxidative stress.

To gain insight into the molecular functions of genes that lie close to CSB occupancy sites, we searched for overlaps with the Molecular Signatures Pathways Database (MSigDB) using the Genomic Regions Enrichment of Annotations Tool (GREAT) (23). The top terms associated with total CSB occupancy in cells treated with menadione involve the roles of gene expression, cell cycle control, spliceosome, and protein metabolism (Supplementary Figure S1B).

We also determined cellular pathways enriched in the list of genes whose promoters are occupied by CSB upon oxidative stress, using the Database for Annotation, Visualization and Integrated Discovery (DAVID) (27,28). The top five KEGG pathways enriched are listed in Supplementary Table S5; they are proteasome, spliceosome, RNA degradation, oxidative phosphorylation and Alzheimer's disease.

### CSB is enriched at CTCF-binding sites upon oxidative stress

**Figure 3.**
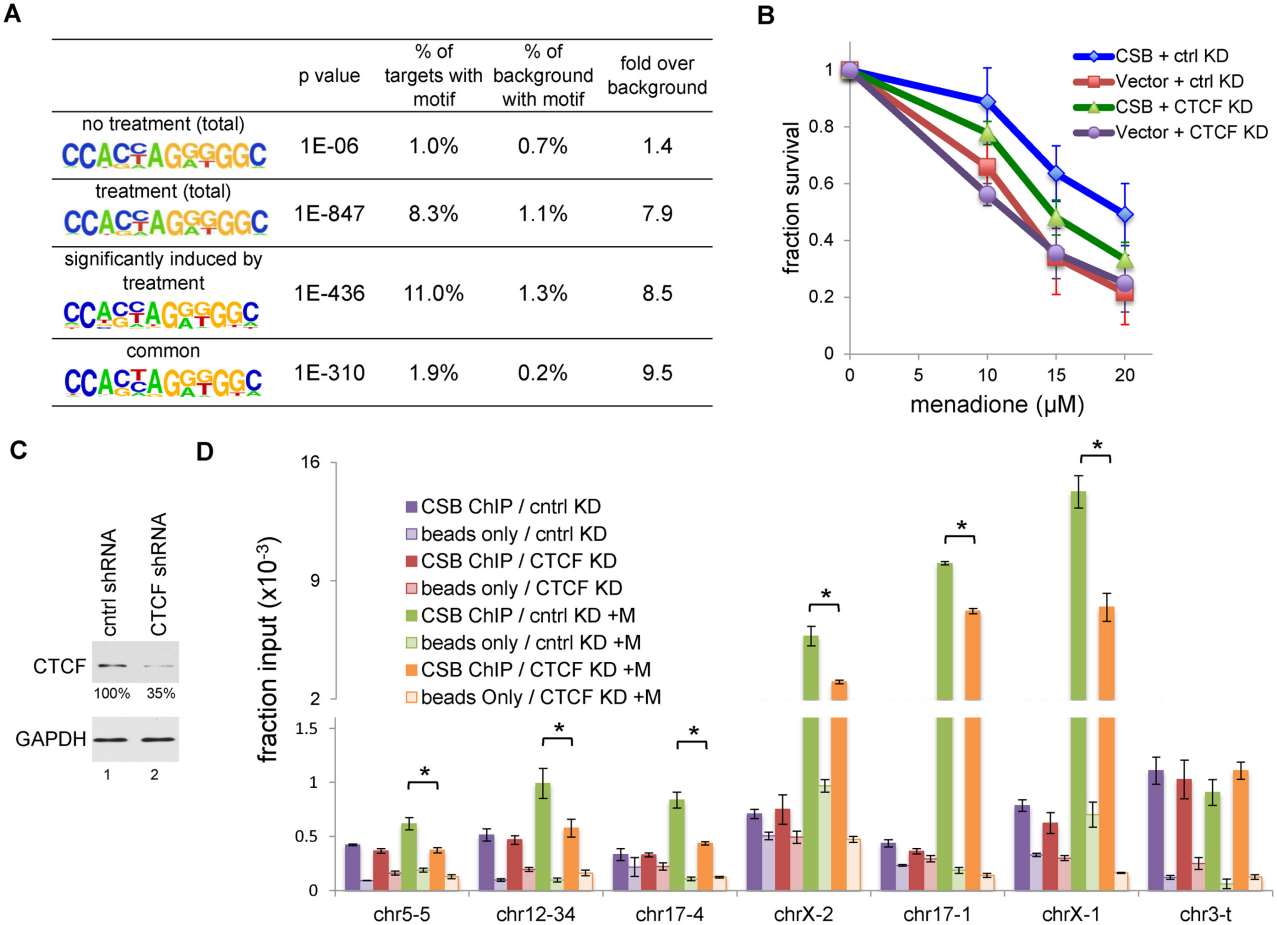
CTCF collaborates with CSB in response to oxidative stress. (**A**) Motif analysis of CSB ChIP-seq data. (**B**) Menadione sensitivity assays on CSB expressing and non-expressing (Vector) cells with decreased CTCF levels. Shown are means ± SEM (*n* = 4). (**C**) Western blot showing a reduction in the CTCF protein level in cells expressing CTCF shRNA. Relative CTCF levels are shown below the CTCF blot. (**D**) CSB ChIP-qPCR assays in cells infected with lentivirus expressing control or CTCF shRNA, with or without with a 1-h menadione treatment (100 μM). Shown are means ± SEM (*n* = 3). A paired *t*-test was used to determine if the difference in CSB enrichment with and without CTCF shRNA treatment was significant. Asterisks indicate *P*-values < 0.05.

To better understand the mechanisms that regulate CSB occupancy at specific genomic regions upon oxidative stress, we used HOMER to identify DNA-binding motifs enriched at CSB occupancy sites (26). As previously reported, CSB was found to occupy c-Jun/AP1-binding sites (9); however, the percentage of CSB-occupied c-Jun/AP1-binding motifs did not change in response to oxidative stress (Supplementary Table S6). Strikingly, CTCF-binding motifs became substantially enriched upon oxidative stress (Figure 3A). In unstressed cells, only 1% of the CSB-occupancy sites contained a CTCF-binding motif, similar to that of the genomic distribution (i.e. background) (Figure 3A). On the other hand, in stressed cells, 8.3% of the CSB-occupancy sites contained CTCF-binding motifs, which is about eight-fold over the background (Figure 3A). Additionally, CTCF-binding motifs are present in 11% of the menadione-induced CSB-occupancy sites (Figure 3A). This observation suggests that CTCF may function with CSB in response to oxidative stress.

To test the hypothesis that CTCF collaborates with CSB to protect cells from oxidative stress, we first determined if cells with decreased CTCF protein levels were more sensitive to ROS. As shown Supplementary Figure S2, cells expressing CTCF shRNA had an approximately 70% reduction in CTCF protein levels as compared to cells expressing a control shRNA. Furthermore, these cells displayed a slight increase in menadione sensitivity as compared to cells expressing a control shRNA (as a paired *t*-test on CSB^wt^ cells with and without CTCF shRNA expression had a *P*-value of 0.08 at 20 μM menadione) (Figure 3B). These results suggest a potential function of CTCF in protecting cells from oxidative stress. Of note, decreasing CTCF levels to 20% in the CSB-null cell line did not further increase ROS sensitivity.

### CTCF regulates a subset of CSB occupancy sites upon oxidative stress

To test the hypothesis that CTCF can alter the genomic occupancy of CSB upon oxidative stress, we selected six sites from our CSB ChIP-seq data that displayed menadione-induced CSB occupancy (Figures 2A and 3D); chr5-5, chr12-34, and chr17-4 contained CTCF-binding motifs while chrX-2, chr17-1, and chrX-1 did not contain CTCF-binding motifs. The chr3-t locus was chosen as a control for ChIP efficiency, since CSB occupancy at this site did not change upon oxidative stress (Figure 3D). ChIP-qPCR confirmed increased CSB occupancy at these six sites in cells treated with menadione as compared to untreated cells.

To test if CTCF contributed to CSB occupancy at these sites, we performed CSB ChIP-qPCR with cells expressing CTCF shRNA (Figure 3C and D). We first confirmed that cells expressing control shRNA and CTCF shRNA had the same amounts of CSB (Supplemental Figure S3). We found that reducing the CTCF protein level by ∼65% selectively reduced CSB occupancy at these sites in menadione treated cells (Figure 3D), indicating that CTCF positively regulates CSB occupancy at these sites upon menadione treatment. Significantly, CTCF knockdown did not decrease basal CSB occupancy. These results together demonstrate that, in response to oxidative stress, CTCF not only regulates CSB occupancy at sites containing CTCF-binding motifs but also at sites devoid of CTCF-binding motifs. Moreover, these results suggest that CTCF may directly interact with CSB.

### Oxidative stress enhances CSB and CTCF interaction

**Figure 4.**
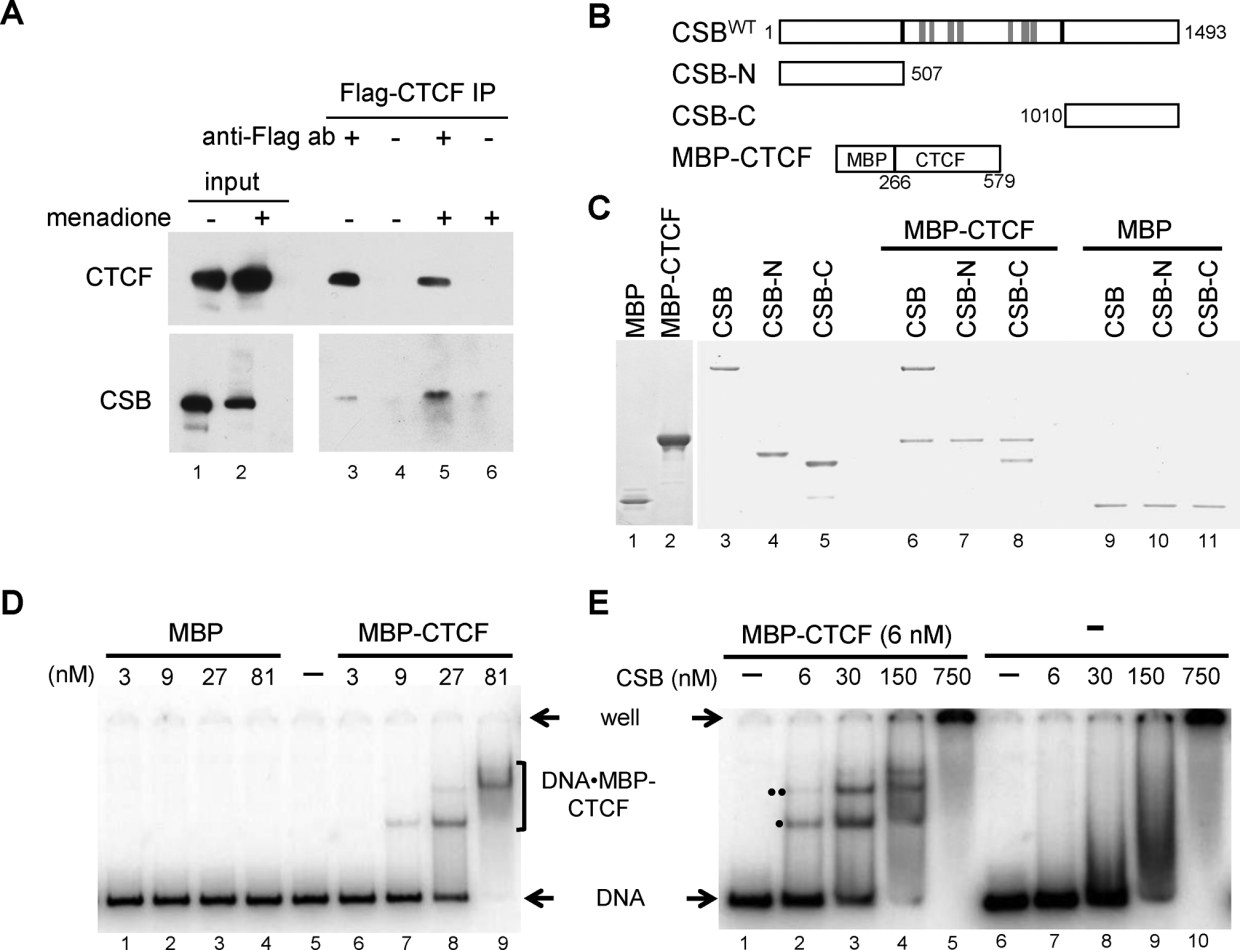
CSB interacts with CTCF in cells and *in vitro*. (**A**) Co-immunoprecipitation of CSB and CTCF in 293T transiently transfected with Flag-tagged CTCF, with or without a 1-h treatment of 100 μM menadione. 3.3% of the lysates used for IP were loaded as input. (**B**) Schematics of recombinant proteins used in (**C**–**E**). All CSB derivatives were N-terminally tagged with the Flag epitope. (C) Coomassie-stained gel showing that CSB directly interacts with CTCF. CSB-C, but not CSB-N, is sufficient for the CTCF association. MBP was used as a negative control. (D and E) EMSA assays showing that CSB enhances CTCF association with DNA. (D) Varying amounts of purified MBP-CTCF (lane 2 in C) or MBP (lane 1 in C) were incubated with a ^32^P-labeled, 200 bp DNA fragment containing a CTCF-binding motif (Supplementary Figure S4B). Protein–DNA complexes were resolved in a native 5% polyacrylamide gel. (E) Varying amounts of purified CSB were incubated with the radiolabeled DNA fragment in the presence or absence of MBP-CTCF. Reactions were subsequently resolved in a 5% native polyacrylamide gel. Protein–DNA complexes marked by ‘•’ and ‘••’ contain the MBP-CTCF protein, as they interacted with an anti-MBP antibody (Supplementary Figure S4A).

To learn more about how CTCF regulates CSB occupancy upon oxidative stress, we determined if CTCF interacts with CSB by co-immunoprecipitation, using lysates prepared from 293T cells expressing a Flag-tagged CTCF protein. As shown in Figure 4A, Flag-CTCF co-immunoprecipitated with CSB, and this interaction increased by about 4-fold after treatment with 100 μM menadione for 1 h. Notably, similar amounts of Flag-CTCF were used in the immunoprecipitation experiments, yet anti-Flag antibodies precipitated less Flag-CTCF from menadione treated cells, suggesting that some of the Flag epitope was occluded in these cells. However, more CSB co-purified with Flag-CTCF in cells treated with menadione, indicating that oxidative stress increases CSB–CTCF interactions in cells.

### CSB directly interacts with CTCF *in vitro*

To determine if CSB and CTCF interact directly, we expressed and purified Flag-tagged CSB and a maltose-binding protein-tagged CTCF derivative (MBP-CTCF), containing the central 11 zinc fingers (aa 269–579) (Figure 4B) (24). As revealed by Coomassie staining, MBP-CTCF directly bound to CSB, while MBP alone did not (Figure 4C, compare lane 6 to 9), indicating that the 11 Zn-fingers of CTCF is sufficient for CSB interaction *in vitro*. Additionally, the C-terminal 483 amino acids of CSB, which lie outside the central catalytic domain, were sufficient for CTCF binding (compare lane 8 to 11). No interaction was detected between the zinc-fingers of CTCF and the first 507 amino acids of CSB (compare lane 7 to 10). Nonetheless, it was previously demonstrated that an endogenously generated CSB-fusion protein, composed of the N-terminal region of CSB fused to a PiggyBac transposase (CSB-PGBD3), was enriched at sites containing CTCF-binding motifs during replicative cell growth (29). Taken together, these observations suggest that CSB and CTCF directly interact and that the interface between these two proteins is multivalent (see discussion) (29).

### CSB positively regulates CTCF-DNA interactions *in vitro*

Given that CSB binds DNA in a sequence-independent manner and CSB directly interacts with CTCF, it is formally possible that CSB may also regulate the interaction of CTCF with DNA (7). To test this hypothesis, we used a 200-bp DNA fragment that contains one perfect, core CTCF-binding site in our *in vitro* protein-DNA binding assays (Figure 4D and E and Supplementary Figure S4B). By incubating MBP-CTCF with radiolabeled DNA, we obtained distinct MBP-CTCF•DNA complexes using electrophoretic mobility shift assays (Figure 4D, lanes 6–9). Distinct complexes were not observed with MBP alone (Figure 4D, lanes 1–4), indicating the protein-DNA complexes in lanes 7–9 were mediated through CTCF.

We next investigated the effect of CSB on the CTCF–DNA interaction. In the presence of 6 nM MBP-CTCF and 1 nM DNA, no clear MBP-CTCF•DNA complex was observed (Figure 4E, lane 1). However, when we included increasing amounts of CSB into the reactions, increasing amounts of DNA-protein complexes were detected (Figure 4E, lanes 2–5). Two observations indicate these DNA-protein complexes contain MBP-CTCF: (i) an anti-MBP antibody can recognize the two bands marked by ‘•’ and ‘••’ (Supplementary Figure S4A), and (ii) given that CSB binds DNA in a sequence-independent manner, DNA-protein complexes containing only CSB would resolve as smears in a native polyacrylamide gel (Figure 4E, lanes 7–10) (7). It is not yet clear why we observed two prominent bands, marked by one and two dots, in the mobility shift assays (Figure 4E). Each of these bands contain the CTCF protein, as they can be super-shifted by and anti-CTCF antibody (Supplementary Figure S4A). Given that two bands appeared in the absence of the CSB protein (Figure 4D), it is unlikely that one of these bands in Figure 4E represents a trimeric CSB–CTCF–DNA complex. Possible explanations for their origin could be different CTCF–DNA stoichiometries, resulting from the binding of a second CTCF protein to an imperfect CTCF-binding site imbedded in the DNA fragment, or different CTCF–DNA conformations, resulting from additional CTCF–DNA contacts that might occur outside of the consensus-binding site. Nonetheless, these results indicate that CSB facilitates the interaction of CTCF with DNA.

### CSB augments CTCF–chromatin interactions in cells

**Figure 5.**
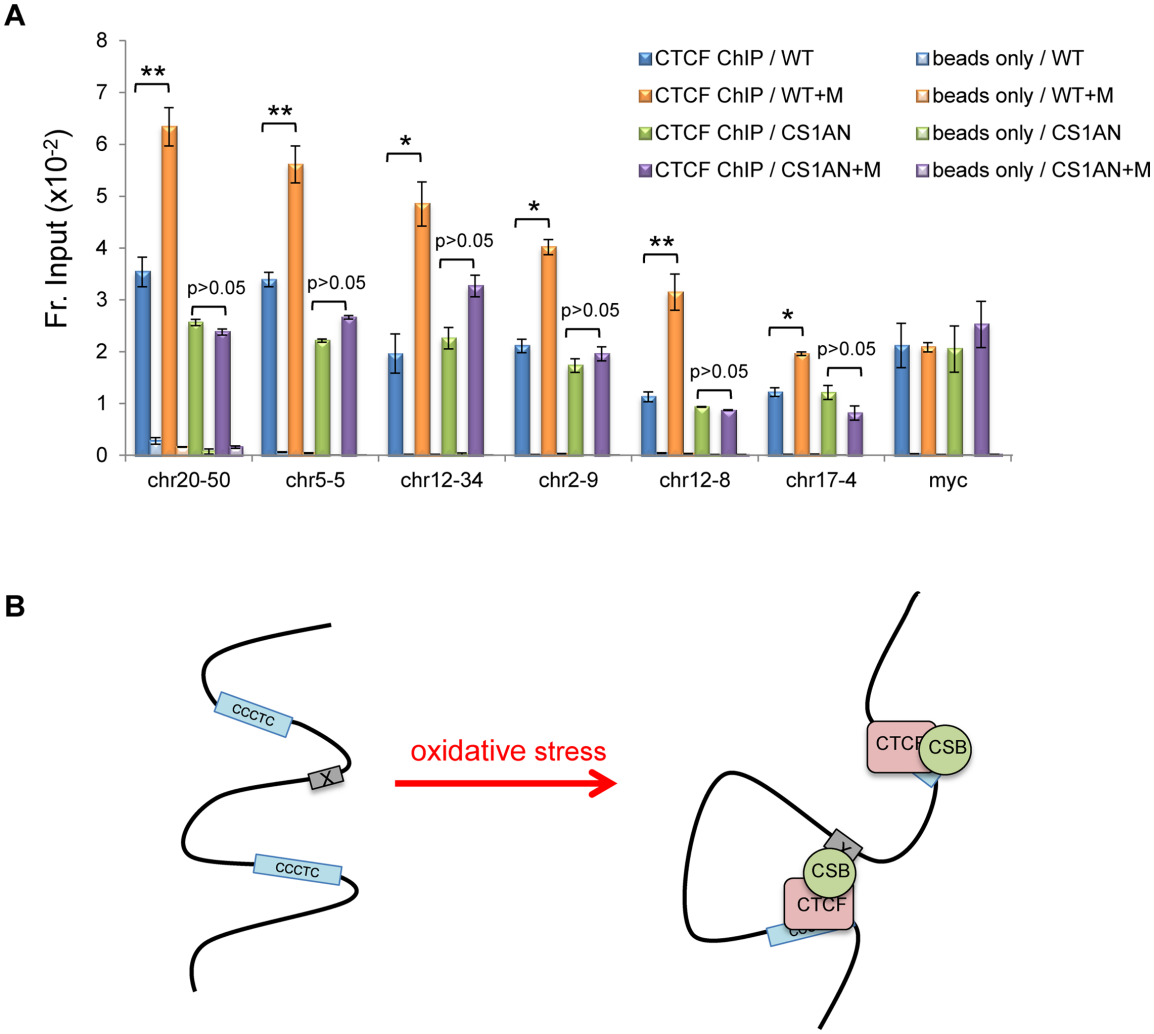
CSB regulates a subset of CTCF occupancy sites upon oxidative stress. (**A**) CTCF ChIP-qPCR assays in CSB expressing (WT) and non-expressing (CS1AN) cells, with or without a 1-h menadione treatment (100 μM). Shown are means ± SEM (*n* = 3). A paired *t*-test was used to determine if the difference in CTCF enrichment before and after menadione treatment was significant. Single asterisks indicate *P* values < 0.05, and double asterisks indicate *P* values < 0.01, as determined by a paired *t*-test. (**B**) Model depicting possible modes of CSB–CTCF chromatin association in response to oxidative stress. CTCF can recruit CSB to CTCF-binding sites or enhance the association of CSB with distal sites. See text for details.

We next used ChIP-qPCR to determine if CSB can regulate the interaction of CTCF with chromatin in cells. We randomly selected six CSB occupied sites that contained CTCF-binding motifs and displayed increased CSB occupancy upon oxidative stress, based on our CSB ChIP-seq data. For these assays, the myc promoter, a known CTCF target, was used to control for CTCF-ChIP efficiency. ChIP assays were performed in CS1AN-sv cells and CS1AN-sv cells reconstituted with CSB^WT^. As shown in Figure 5A, we observed a significant increase in CTCF occupancy at chr20-50, chr5-5, chr12-34, chr2-9, chr12-8, and chr17-4 in a menadione-dependent manner in CSB^WT^ cells. Strikingly, in cells without CSB (CS1AN-sv cells), we did not observe significant menadione-dependent changes in CTCF occupancy at these six sites. No changes were observed for CTCF occupancy at the myc promoter.

## DISCUSSION

Previously, we found that CSB is enriched at genomic regions containing epigenetic features of enhancers and promoters during replicative cell growth (9). We also found that CSB can alter chromatin structure near its occupancy site to regulate transcription (9). In this study, we found a dramatic increase in CSB occupancy at promoters upon oxidative stress: about 20% of the CSB occupancy sites that are induced in oxidatively stressed cells lie in promoter regions as compared to the genomic distribution of promoters, which is only 1%, supporting a function of CSB in transcription regulation upon oxidative stress (Figure 2). The top terms associated with total sites of CSB occupancy in cells treated with menadione involve the roles of gene expression, cell cycle control, spliceosome, and protein metabolism (Supplementary Figure S1B), suggesting that CSB might play a general role in regulating RNA and protein homeostasis as well as cell division in response to oxidative stress. Pathway analysis of the genes with their promoter regions occupied by CSB upon oxidative stress suggests that CSB might also control energy and ROS production by regulating the oxidative phosphorylation machinery at the transcriptional level (Supplementary Table S5). Indeed, defects in mitochondrial function have been associated with cells lacking functional CSB (30). Of note, we currently cannot exclude the possibility that 100 μM menadione might induce a CSB response that is not only related to the relief of oxidative stress but also to those related to cell death, resulting from excessive oxidative stress. Future ChIP-seq studies examining CSB occupancy in response to different menadione doses will help to distinguish between these different CSB functions.

CSB has been suggested to participate in the repair of oxidized bases (31–34), and this study does not exclude this possibility. Oxidized DNA lesions would be, to a large degree, randomly distributed throughout the genome, and the association of CSB with oxidized DNA would, therefore, not resolve as defined anti-CSB ChIP-seq peaks.

We have shown that CTCF directly interacts with CSB and impacts CSB occupancy at specific genomic regions upon oxidative stress (Figure 3), revealing a novel mechanism by which the activity of this chromatin remodeler can be regulated. Although we have identified sites which contain the CTCF-binding motif, and to which CSB demonstrated CTCF-dependent occupancy (chr5-5, chr12-34 and chr17-4, Figure 3D), the level of CSB occupancy at these sites is 5–15-fold less than that of CSB at the chrX-2, chr17-1 and chrX-1 loci, which do not contain a CTCF-binding motif. These observations suggest that CSB is recruited to the latter sites through another mechanism. Remarkably, we also observed CTCF-dependent CSB enrichment at sites without CTCF-binding motifs, such as chrX-2, chr17-1 and chrX-1, suggesting that CTCF may stabilize CSB occupancy at these sites. Taken together, the physical and functional interaction between CSB and CTCF that is greatly enhanced upon oxidative stress may have the potential to establish DNA loops to regulate gene expression in response to oxidative stress (Figure 5B).

Another transcript originating from the CSB locus generates a protein composed of the N-terminal 465 residues of CSB fused to a piggyBac transposase (CSB-PGBD3) (35). Strikingly, CSB-PGBD3 was found enriched at sites containing CTCF-binding motifs (29). However, the association of CSB-PGBD3 with CTCF-binding sites is different from that of CSB, as it occurs in the absence of oxidative stress (29). The results of that study suggested that the N-terminal region of CSB could interact with CTCF and that CTCF and CSB-PGBD3 may play roles in chromosomal looping during replicative cell growth. Given that we did not see a direct interaction between the N-terminal 507 residues of CSB and the 11 Zn-fingers of CTCF (Figure 4), these results suggest that CSB-N likely interacts with full-length CTCF or a region that flanks the central CTCF Zn-finger domain. During replicative cell growth, the N-terminal region of CSB occludes a chromatin interaction surface in the C-terminal region (20). This occlusion might, in part, explain why CSB-PGBD3 association with CTCF-binding motifs occurs in the absence of stress, while the association of CSB with CTCF-binding motifs preferentially occurs upon oxidative stress.

Collectively, these observations suggest that CSB and CTCF have at least two regions of contact. Therefore, long-range chromosomal interactions that might be mediated by CSB and CTCF (Figure 5B) may be asymmetric, with the central CTCF Zn-finger domain binding to a CTCF motif at one end, CSB binding to a regulatory site at the other end, and a strong protein bridge between the ends mediated by two interactions: one between the CTCF zinc finger domain and the CSB C-terminal region, and the other between part or all of CTCF and the CSB N-terminal region. Moreover, such looping would be further reinforced as CSB and CTCF can, reciprocally, stabilize each other's binding to DNA: CSB can enhance CTCF binding to a CTCF motif *in vitro* (Figure 4D-E), menadione strongly induces the CSB-CTCF interaction in cells (Figure 4A), and even a modest CTCF knockdown (∼65%) can reduce menadione-inducible CSB binding to sites that lack a CTCF motif (Figure 3D).

The basis for the oxidative stress-enhanced CSB-CTCF interaction remains to be determined. Change in post-translational modification is one possibility, as stress-associated changes have been observed for both CSB and CTCF (36–38). For example, upon oxidative stress, CSB has been suggested to be poly(ADP-ribosyl)ated and phosphorylated, and CTCF is found to be de-sumoylated.

The only other chromatin remodeler that has been shown to interact with CTCF is the chromodomain–helicase–DNA-binding protein 8 (CHD8), and this interaction is critical for CTCF-dependent insulator function (39). Intriguingly, CHD8 also associates with the zinc-fingers of CTCF, as do several other proteins (e.g. Sin3A and YB-1), indicating that the zinc-finger region of CTCF can support both protein–DNA and protein–protein interactions (39–41). Although all 11 zinc-fingers of CTCF could, in principle, associate with DNA, a CTCF–DNA association requires only a subset of zinc-fingers (42,43). Therefore, some of the CTCF–zinc-fingers may be free to interact with proteins, such as CSB. Indeed, the consensus CTCF sequence that was recovered from our ChIP-seq analysis contained only the core CTCF-binding site. Nonetheless, whether or not a specific CTCF-binding site in the genome can allow for both CTCF–DNA and CTCF–CSB interactions may depend upon the number of DNA–CTCF Zn-finger contacts made at that site (42), as well as the relative affinities of specific CTCF Zn-fingers for DNA versus CSB.

CTCF plays a fundamental role in organizing long-range chromatin structure (19). Chromosome conformation capture-based studies have revealed that chromatin fibers can be organized into different topologically associating domains, termed TADs. Strikingly, ∼85% of CTCF occupancy sites lie within TADs and ∼15% lie at TAD borders. CTCF has long been known to function in transcriptional activation as well as repression. One current model to explain the multiple CTCF functions is that CTCF promotes interactions between transcription regulatory elements within a TAD and precludes interactions between regulatory elements of different TADs (19). More recently, Li *et al*. have shown that the heat shock response can cause widespread rearrangement of 3D chromatin organization and lower the CTCF occupancy at the boundaries of TADs, leading to a decrease in intra-TAD interactions and an increase in new inter-TAD interactions (44).

It remains to be determined how CSB associates with only a subset of CTCF binding sites. Regardless of the mechanism that imparts CTCF-binding site specificity, our observation that CSB can regulate CTCF-DNA interactions *in vitro* and in cells supports a hypothesis that CSB and CTCF can reciprocally regulate each other's interactions with chromatin, leading to the establishment of new, long-range chromosome associations upon oxidative stress. It is clear that modulating the association of CTCF with chromatin can have profound impacts on chromatin organization, which in turn can influence fundamental processes, such as transcription. Accordingly, we would like to speculate that the ATP-dependent chromatin remodeler CSB cooperates with CTCF to protect cells from oxidative stress by regulating long-range chromosomal interactions. Future experiments using chromatin conformation capture-based approaches will offer more insights into the functions of the CSB-CTCF collaboration during oxidative stress.

## Supplementary Material

SUPPLEMENTARY DATA
