# Supplementary material for: The CSB chromatin remodeler and CTCF architectural protein cooperate in response to oxidative stress
Source: Nucleic Acids Res. 2015 Nov 17;44(5):2125–35. doi: 10.1093/nar/gkv1219 (PMC4797267; doi:10.1093/nar/gkv1219)
Supplement: SUPPLEMENTARY DATA [file supp_44_5_2125__index.html]

The CSB chromatin remodeler and CTCF architectural protein cooperate in response to oxidative stress — SUPPLEMENTARY DATA 

# The CSB chromatin remodeler and CTCF architectural protein cooperate in response to oxidative stress

## SUPPLEMENTARY DATA

- SUPPLEMENTARY DATA
- SUPPLEMENTARY DATA
