# Supplementary material for: The CSB chromatin remodeler and CTCF architectural protein cooperate in response to oxidative stress
Source: Nucleic Acids Res. 2015 Nov 17;44(5):2125–35. doi: 10.1093/nar/gkv1219 (PMC4797267; doi:10.1093/nar/gkv1219)

Supplementary Figure Legends

Figure S1. Genomic distributions and gene ontology of CSB occupancy sites. (A) Total CSB occupancy sites in cells untreated or treated with menadione were analyzed for their genomic distribution. (B) MSigDB pathways analysis of total CSB occupancy sites in cells treated with menadione.

Figure S2. Western blot analysis of CTCF levels in cells used in Figure 3B. Significant reduction in CTCF protein levels were achieved by shRNA treatment in cells without functional CSB (vector) or reconstituted with CSB (CSB).

Figure S3. CSB protein levels in shRNA-expressing cells. Whole cell lysates from cells treated for 5 days with a control shRNA or CTCF shRNA were resolved by SDS-PAGE. Shown are western blots using antibodies that recognize CTCF, CSB and GAPDH.

Figure S4. Antibody supershift assays. (A) EMSA assays were used to visualize supershifts of MBP-CTCF•DNA complexes, in the presence or absence of CSB. The monoclonal anti-MBP antibody was added to reactions as indicated. The anti-MBP antibody recognizes both protein-DNA complexes marked by "•" and "••", indicating that both protein-DNA complexes contain MBP-CTCF. (B) DNA sequences used in the assays. The CTCF motif is underlined.

Figure S5. CTCF protein levels in CS1AN cells and CS1AN cells reconstituted with CSB. Whole cell lysate from CS1AN cells and CS1AN reconstituted with CSB were resolved by

SDS-PAGE. Shown are western blot analyses using antibodies that recognize CSB, CTCF and GAPDH.

Supplementary Tables

Table S1: Sequence reads summary of CSB ChIP-seq data

Table S2: CSB occupancy unique to cells treated with menadione

Table S3: CSB occupancy common to cells with or without menadione treatment

Table S4: CSB occupancy unique to cells without menadione treatment

Table S5: KEGG pathway analysis of promoters occupied by CSB upon oxidative stress

Table S6: Enrichment of Jun/AP1 motifs at sites of CSB occupancy

Table S7: Primers used in qPCR assays.

Lake_SFig 1

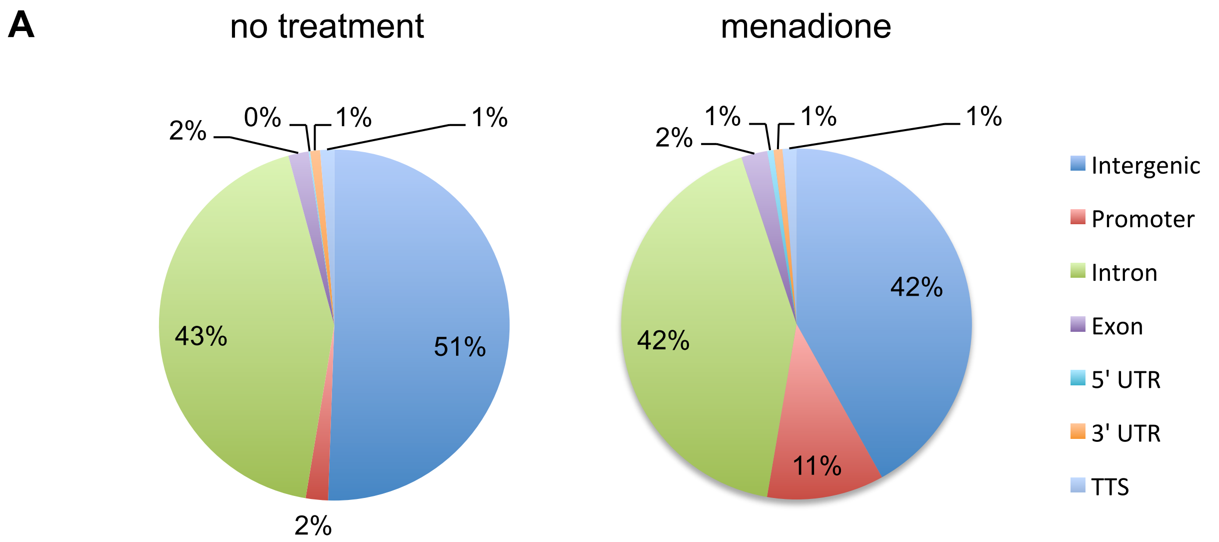
A
no treatment
menadione
2%
0%
1%
1%
43%
51%
2%
2%
1%
1%
1%
42%
42%
11%
Intergenic
Promoter
Intron
Exon
5' UTR
3' UTR
TTS

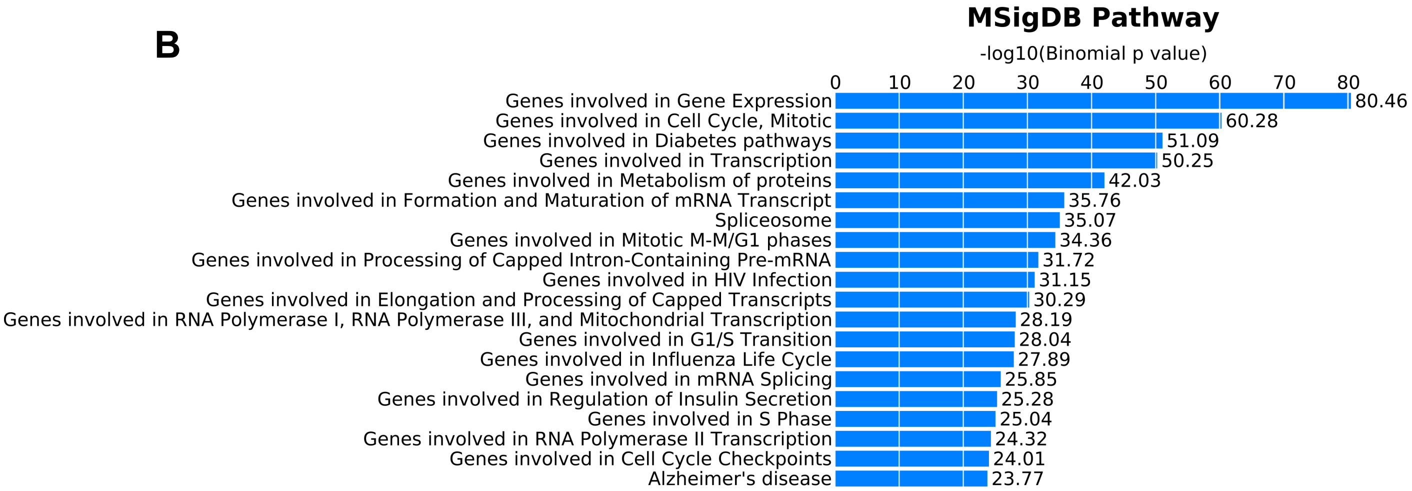
B
MSigDB Pathway
-log10(Binomial p value)
0
10
20
30
40
50
60
70
80
Genes involved in Gene Expression 80.46
Genes involved in Cell Cycle, Mitotic 60.28
Genes involved in Diabetes pathways 51.09
Genes involved in Transcription 50.25
Genes involved in Metabolism of proteins 42.03
Genes involved in Formation and Maturation of mRNA Transcript 35.76
Spliceosome 35.07
Genes involved in Mitotic M-M/G1 phases 34.36
Genes involved in Processing of Capped Intron-Containing Pre-mRNA 31.72
Genes involved in HIV Infection 31.15
Genes involved in Elongation and Processing of Capped Transcripts 30.29
Genes involved in RNA Polymerase I, RNA Polymerase III, and Mitochondrial Transcription 28.19
Genes involved in G1/S Transition 28.04
Genes involved in Influenza Life Cycle 27.89
Genes involved in mRNA Splicing 25.85
Genes involved in Regulation of Insulin Secretion 25.28
Genes involved in S Phase 25.04
Genes involved in RNA Polymerase II Transcription 24.32
Genes involved in Cell Cycle Checkpoints 24.01
Alzheimer's disease 23.77

Lake_SFig2

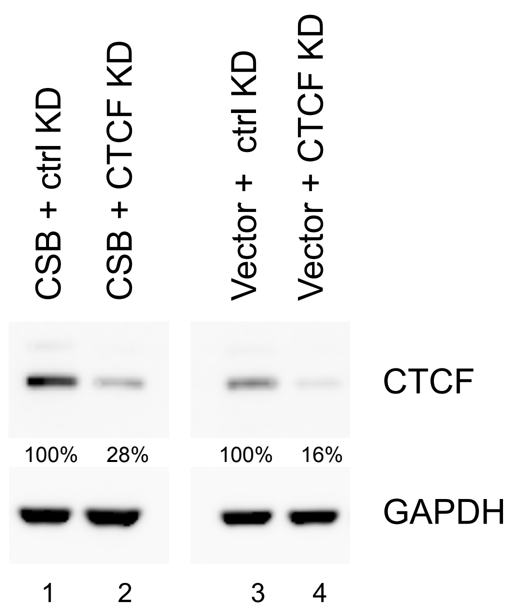
CSB + ctrl KD
CSB + CTCF KD
Vector + ctrl KD
Vector + CTCF KD
CTCF
100%
28%
100%
16%
GAPDH
1
2
3
4

Lake_SFig.3

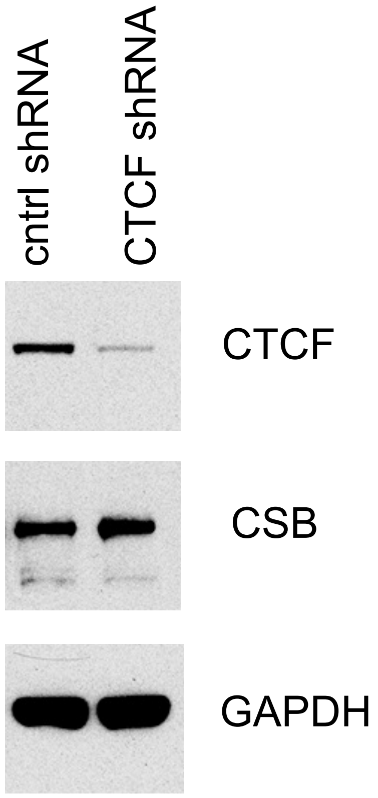

Lake_SFig4

A

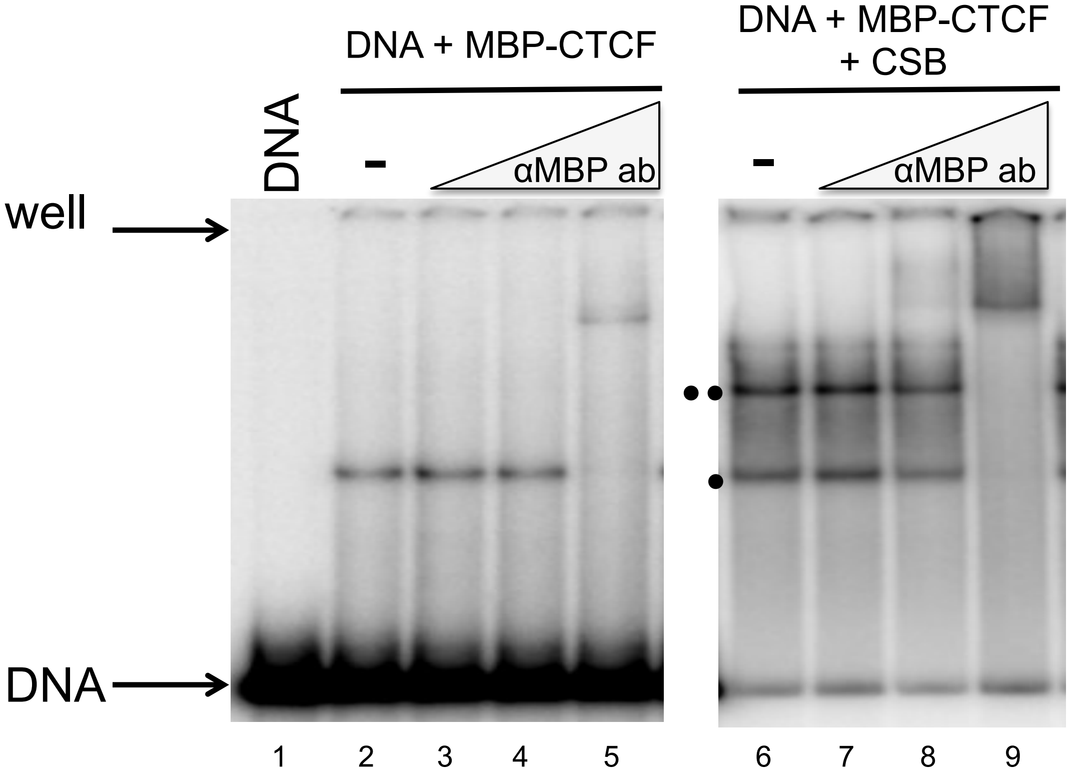

B

CACACCGAGT TCATCCCTTA TGTGATGGAC CCTCCACGAG GGGGCCCTGG
AGAATCCCGG TGCCGAGGCT GCAGAATTGG TCGTAGACAG CTCTAGCACC
GCTTAAACGC ACGTACGCGC TGTCCCCCGC GTTTTAACCG CCAAGGGGAT
TACTCCCTAG TCTCCAGGCA CGTGTCAGAT ATATACATCC TGT

Lake_SFig.5

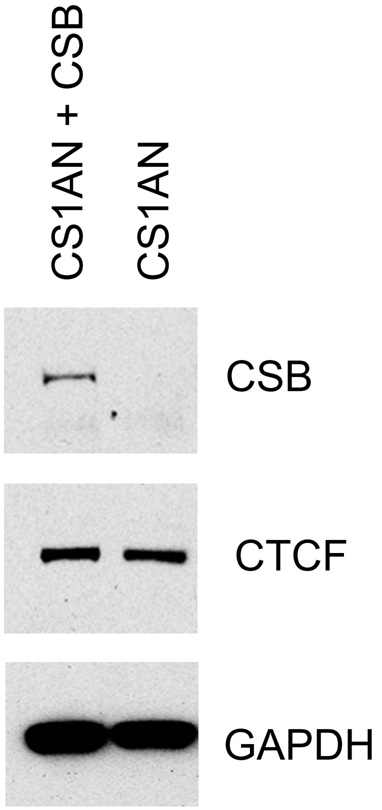

Supplement: SUPPLEMENTARY DATA [file supp_gkv1219_nar-02394-d-2015-File007.pdf]
